# Crystal structure of 2-amino-5-nitro­pyridinium sulfamate

**DOI:** 10.1107/S2056989015000365

**Published:** 2015-01-31

**Authors:** M. Ambrose Rajkumar, M. NizamMohideen, S. Stanly John Xavier, S. Anbarasu, Dr. Prem Anand Devarajan

**Affiliations:** a Physics Research Centre, Department of Physics, St. Xavier’s College (Autonomous), Palayamkottai 627 002, Tamil Nadu, India; b Department of Physics, The New College (Autonomous), Chennai 600 014, Tamil Nadu, India; c Department of Chemistry, St. Xavier’s College (Autonomous), Palayamkottai 627 002, Tamil Nadu, India

**Keywords:** crystal structure, sulfamic acid, 2-amino-5-nitro­pyridine, sulfamate, 2-amino-5-pyridinium, mol­ecular salt, hydrogen bonding.

## Abstract

The title mol­ecular salt, C_5_H_6_N_3_O_2_^+^ ·H_2_NO_3_S^−^, was obtained from the reaction of sulfamic acid with 2-amino-5-nitro­pyridine. A proton transfer from sulfamic acid to the pyridine N atom occurred, resulting in the formation of a salt. As expected, this protonation leads to the widening of the C—N—C angle of the pyridine ring, to 122.9 (3)°, with the pyridinium ring being essentially planar (r.m.s. deviation = 0.025 Å). In the crystal, the ion pairs are joined by three N—H⋯O and one N—H⋯N hydrogen bonds in which the pyridinium N atom and the amino N atom act as donors, and are hydrogen bonded to the carboxyl­ate O atoms and the N atom of the sulfamate anion, thus generating an *R* ^3^_3_(22) ring motif. These motifs are linked by further N—H⋯O hydrogen bonds enclosing *R* ^3^_3_(8) loops, forming sheets parallel to (100). The sheets are linked *via* weak C—H⋯O hydrogen bonds, forming a three-dimensional structure. The O atoms of the nitro group are disordered over two sets of sites with a refined occupancy ratio of 0.737 (19):0.263 (19).

## Abstract 2

The title mol­ecular salt, obtained by the reaction of sulfamic acid with 2-amino-5-nitro­pyridine, is the result of a proton transfer from sulfamic acid to the N atom of the pyridine ring. In the crystal, the cations and anions are linked by a number of N—H⋯O and N—H⋯N hydrogen bonds, forming sheets lying parallel to (100).

## Chemical context   

Pyridine heterocycles and their derivatives are present in many large mol­ecules having photo-chemical, electro-chemical and catalytic applications. Some pyridine derivatives possess non-linear optical (NLO) properties (Babu *et al.*, 2014*a* ▸,*b* ▸). Simple organic–inorganic salts containing strong inter­molecular hydrogen bonds have attracted attention as materials which display ferroelectric–paraelectric phase transitions (Sethuram, *et al.*, 2013*a* ▸,*b* ▸; Huq *et al.*, 2013 ▸; Shihabuddeen Syed *et al.*, 2013 ▸; Showrilu *et al.*, 2013 ▸). We have recently reported the crystal structures of 2-amino-6-methyl­pyridinium 2,2,2-tri­chloro­acetate (Babu *et al.*, 2014*a* ▸), 2-amino-6-methyl­pyridinium 4-methyl­benzene­sulfonate (Babu *et al.*, 2014*b* ▸) and 2-amino-5-nitro­pyridinium hydrogen oxalate (Rajkumar *et al.*, 2014 ▸). In a continuation of our studies of pyridinium salts, we report herein on the crystal structure of the title mol­ecular salt, obtained by the reaction of 2-amino-5-nitro­pyridine with sulfamic acid.
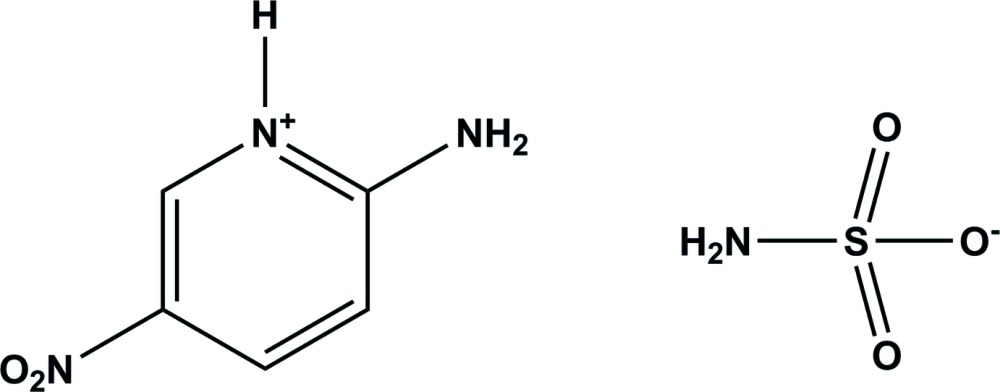


## Structural commentary   

**Figure 1:**
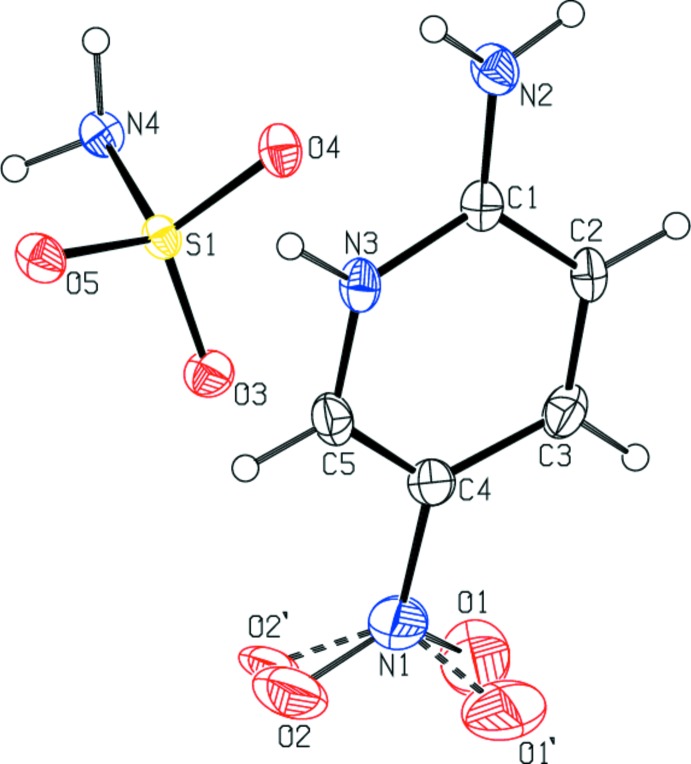
View of the mol­ecular structure of the title mol­ecular salt, with atom labelling. Displacement ellipsoids are drawn at the 50% probability level.

The asymmetric unit of the title compound, Fig. 1 ▸, consists of a 2-amino-5-nitro­pyridin-1-ium cation and a sulfamate anion. The bond lengths and angles are within normal ranges and comparable with those in closely related structures (Babu *et al.*, 2014*a* ▸,*b* ▸; Rajkumar *et al.*, 2014 ▸). A proton transfer from the sulfamic acid to the pyridine atom N3 resulted in the formation of a salt. This protonation leads to the widening of the C5—N3—C1 angle of the pyridine ring to 122.9 (3)°, compared with 115.25 (13)° in unprotonated amino­pyridine (Anderson *et al.*, 2005 ▸). This type of protonation is observed in various amino­pyridine acid complexes (Babu *et al.*, 2014*a* ▸,*b* ▸; Rajkumar *et al.*, 2014 ▸). In the sulfamate anion the S—O distances vary from 1.440 (3) to 1.460 (2) Å, and O—S—O angles vary from 111.59 (15) to 114.22 (15) °.

In the cation, the N2—C1 [1.317 (5) Å] bond is shorter than the N3—C1 [1.357 (4) Å] and N3—C5 [1.340 (5) Å] bonds, and the C1—C2 [1.411 (5) Å] and C3—C4 [1.402 (6) Å] bonds lengths are significantly longer than bonds C2—C3 [1.348 (5) Å] and C4—C5 [1.338 (6) Å], similar to those observed previously for the amino­pyridinium cation (Babu *et al.*, 2014*a* ▸,*b* ▸; Rajkumar *et al.*, 2014 ▸). In contrast, in the solid-state structure of amino­pyridinium, the C—N(H_2_) bond is clearly longer than that in the ring (Nahringbauer & Kvick, 1977 ▸). The geometrical features of the amino­pyridinium cation (N1/N3/C1–C5) resemble those observed in other 2-amino­pyridinium structures (Babu *et al.*, 2014*a* ▸,*b* ▸; Rajkumar *et al.*, 2014 ▸) that are believed to be involved in amine–imine tautomerism (Ishikawa *et al.*, 2002 ▸). However, previous studies have shown that a pyridinium cation always possesses an expanded C—N—C angle in comparison with pyridine itself (Jin *et al.*, 2005 ▸).

In this atomic arrangement, one can distinguish the inter­cation-to-anion contact C5—H5⋯O3 (H5⋯O5 = 2.41 Å), which induces the aggregation of the independent organic cation 2-amino-5-nitro­pyridinium. This kind of arrangement is also observed in the related structure of 2-amino-5-nitro­pyridinium hydrogen selenate (Akriche & Rzaigui, 2009 ▸). These pairs are located between the anionic layers to link them by various inter­actions. The geometric features of the organic cation are usual and comparable with values observed for other 2-amino nitro­pyridinium compounds (Akriche & Rzaigui, 2009 ▸). It is worth noticing that the C—NH_2_ [1.317 (5) Å] and C—NO_2_ [1.449 (6) Å] distances in the cations are, respectively, shortened and lengthened with respect to the same bond lengths [1.337 (4) and 1.429 (4) Å] observed for 2-amino-nitro­pyridine (Aakeroy *et al.*, 1998 ▸). All the 2-amino-nitro­pyridinium cations encapsulated in various anionic sub-networks show the same changes in the C—NH_2_ and C—NO_2_ distances, revealing a weak increase of π bond character in the bond C—NH_2_ and a decrease in the bond C—NO_2_.

## Supra­molecular features   

**Figure 2:**
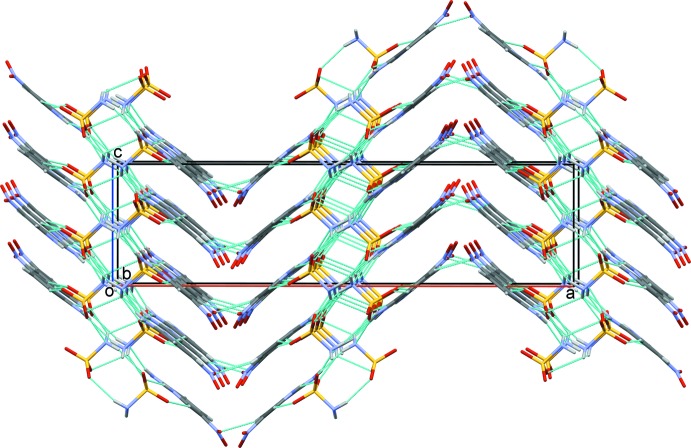
The crystal packing of the title salt, viewed along the *b* axis. The hydrogen bonds are shown as dashed lines (see Table 1 ▸ for details; only the major components of the disordered nitro O atoms are shown).

In the crystal, the ion pairs are linked by the N—H⋯O and N—H⋯N hydrogen bonds (Table 1 ▸ and Fig. 2 ▸). The proton­ated atom (N3) and the 2-amino group (N2) of the cation are hydrogen bonded to the carboxyl­ate oxygen atoms (O5 and O4) and the nitro­gen atom (N4) of the sulfamate anion *via* a pair of N—H⋯O and N—H⋯N (N3—H3*A*⋯O5, N2—H2*B*⋯O4 and N2—H2*A*⋯N4) hydrogen bonds (Table 1 ▸), forming an 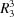
(22)ring motif. These motifs are further linked by N—H⋯O hydrogen bonds, enclosing 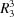
(8) loops, and forming sheets lying parallel to (100). Weak C—H⋯O hydrogen bonds link the sheets, forming a three-dimensional structure (Fig. 2 ▸ and Table 1 ▸). The identification of such supra­molecular patterns will help us design and construct preferred hydrogen-bonding patterns of drug-like mol­ecules.

## Database survey   

A search of the Cambridge Structural Database (CSD, Version 5.35, May 2014; Groom & Allen, 2014 ▸) for the cation 2-amino-5-nitro­pyridinium gave 42 hits for which there were 36 hits with atomic coordinates present. For these structures, the average C—N—C bond angle is *ca* 123°, while the average C—N(H_2_) and C—N(O_2_) bond lengths are *ca* 1.32 and 1.45 Å, respectively. A search for the anion amino­sulfamate gave 23 hits but only 17 contained atomic coordinates. Here the S—O bond lengths vary from *ca* 1.399 to 1.469 Å, while the N—S bond length varies from *ca* 1.63 to 1.80 Å. The bond lengths and angles in the title salt are very similar to those reported for the various structures in the CSD.

## Synthesis and crystallization   

The starting material 2-amino-5-nitro­pyridine was obtained by treating 3-nitro­pyridine with ammonia in the presence of KMnO_4_. Colourless block-like crystals of the title salt were obtained by slow evaporation of a 1:1 equimolar mixture of 2-amino-5-nitro­pyridine and sulfamic acid in methanol at room temperature.

## Refinement   

Crystal data, data collection and structure refinement details are summarized in Table 2 ▸. The N-bound H atoms were located in a difference Fourier map and refined with distance restraints: N—H = 0.89 (2) Å. The C-bound H atoms were positioned geometrically and refined using a riding model: C—H = 0.93 Å with *U*_iso_(H) = 1.2*U*_eq_(C). The O atoms of the nitro group are disordered over two sets of sites (O1/O1′ and O2/O2′) with a refined occupancy ratio of 0.737 (19):0.263 (19).

## Supplementary Material

Crystal structure: contains datablock(s) global, I. DOI: 10.1107/S2056989015000365/su5048sup1.cif

Structure factors: contains datablock(s) I. DOI: 10.1107/S2056989015000365/su5048Isup2.hkl

Supporting information file. DOI: 10.1107/S2056989015000365/su5048Isup3.cml

CCDC reference: 1042506

Additional supporting information:  crystallographic information; 3D view; checkCIF report

## Figures and Tables

**Table 1:** Hydrogen-bond geometry (, )

*D*H*A*	*D*H	H*A*	*D* *A*	*D*H*A*
N2H2*B*O4^i^	0.88(2)	1.98(2)	2.861(4)	176(4)
N2H2*A*N4^ii^	0.88(2)	2.18(2)	3.044(4)	169(4)
N3H3*A*O5^iii^	0.89(2)	1.91(2)	2.766(4)	163(4)
N4H4*B*O4^iv^	0.89(2)	2.20(2)	3.073(4)	166(3)
N4H4*A*O5^v^	0.89(2)	2.20(2)	2.960(4)	143(3)
C2H2O3^i^	0.93	2.57	3.469(4)	163
C3H3O2^vi^	0.93	2.46	3.328(13)	155
C5H5O3^iii^	0.93	2.41	3.187(4)	141

Symmetry codes: (i) 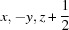; (ii) 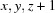; (iii) 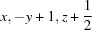; (iv) 
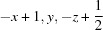
; (v) 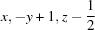; (vi) 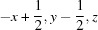.

**Table 2:** Experimental details

Crystal data	
Chemical formula	C_5_H_6_N_3_O_2_^+^H_2_NO_3_S
*M*_r_	236.21
Crystal system, space group	Orthorhombic, *P* *b* *c* *n*
Temperature (K)	293
*a*, *b*, *c* ()	28.0866(10), 9.0052(3), 7.4023(2)
*V* (^3^)	1872.23(10)
*Z*	8
Radiation type	Mo *K*
(mm^1^)	0.36
Crystal size (mm)	0.35 0.30 0.25

Data collection	
Diffractometer	Bruker Kappa APEXII CCD
Absorption correction	Multi-scan (*SADABS*; Bruker, 2004 ▸)
*T*_min_, *T*_max_	0.887, 0.917
No. of measured, independent and observed [*I* > 2(*I*)] reflections	15358, 1653, 1557
*R*_int_	0.024
(sin /)_max_ (^1^)	0.594

Refinement	
*R*[*F* ^2^ > 2(*F* ^2^)], *wR*(*F* ^2^), *S*	0.055, 0.111, 1.28
No. of reflections	1653
No. of parameters	175
No. of restraints	50
H-atom treatment	H atoms treated by a mixture of independent and constrained refinement
_max_, _min_ (e ^3^)	0.27, 0.45

Computer programs: *APEX2*, *SAINT* and *XPREP* (Bruker, 2004 ▸), *SHELXS97* and *SHELXL97* (Sheldrick, 2008 ▸), *ORTEP-3 for Windows* and *WinGX* (Farrugia, 2012 ▸), *Mercury* (Macrae *et al.*, 2008 ▸) and *PLATON* (Spek, 2009 ▸).

## supplementary crystallographic information

### Crystal data

**Table:**

C_5_H_6_N_3_O_2_^+^·H_2_NO_3_S^−^	*F*(000) = 976
*M**_r_* = 236.21	*D*_x_ = 1.676 Mg m^−^^3^
Orthorhombic, *P**b**c**n*	Mo *K*α radiation, λ = 0.71073 Å
Hall symbol: -P 2n 2ab	Cell parameters from 1653 reflections
*a* = 28.0866 (10) Å	θ = 2.4–31.1°
*b* = 9.0052 (3) Å	µ = 0.36 mm^−^^1^
*c* = 7.4023 (2) Å	*T* = 293 K
*V* = 1872.23 (10) Å^3^	Block, colourless
*Z* = 8	0.35 × 0.30 × 0.25 mm

### Data collection

**Table:**

Bruker Kappa APEXII CCD diffractometer	1653 independent reflections
Radiation source: fine-focus sealed tube	1557 reflections with *I* > 2σ(*I*)
Graphite monochromator	*R*_int_ = 0.024
ω and φ scans	θ_max_ = 25.0°, θ_min_ = 2.4°
Absorption correction: multi-scan (*SADABS*; Bruker, 2004)	*h* = −33→33
*T*_min_ = 0.887, *T*_max_ = 0.917	*k* = −10→10
15358 measured reflections	*l* = −8→8

### Refinement

**Table:**

Refinement on *F*^2^	Primary atom site location: structure-invariant direct methods
Least-squares matrix: full	Secondary atom site location: difference Fourier map
*R*[*F*^2^ > 2σ(*F*^2^)] = 0.055	Hydrogen site location: inferred from neighbouring sites
*wR*(*F*^2^) = 0.111	H atoms treated by a mixture of independent and constrained refinement
*S* = 1.28	*w* = 1/[σ^2^(*F*_o_^2^) + (0.0116*P*)^2^ + 5.4481*P*] where *P* = (*F*_o_^2^ + 2*F*_c_^2^)/3
1653 reflections	(Δ/σ)_max_ < 0.001
175 parameters	Δρ_max_ = 0.27 e Å^−^^3^
50 restraints	Δρ_min_ = −0.44 e Å^−^^3^

### Special details

**Table:**

Geometry. All e.s.d.'s (except the e.s.d. in the dihedral angle between two l.s. planes) are estimated using the full covariance matrix. The cell e.s.d.'s are taken into account individually in the estimation of e.s.d.'s in distances, angles and torsion angles; correlations between e.s.d.'s in cell parameters are only used when they are defined by crystal symmetry. An approximate (isotropic) treatment of cell e.s.d.'s is used for estimating e.s.d.'s involving l.s. planes.
Refinement. Refinement of *F*^2^ against ALL reflections. The weighted *R*-factor *wR* and goodness of fit *S* are based on *F*^2^, conventional *R*-factors *R* are based on *F*, with *F* set to zero for negative *F*^2^. The threshold expression of *F*^2^ > σ(*F*^2^) is used only for calculating *R*-factors(gt) *etc*. and is not relevant to the choice of reflections for refinement. *R*-factors based on *F*^2^ are statistically about twice as large as those based on *F*, and *R*- factors based on ALL data will be even larger.

### Fractional atomic coordinates and isotropic or equivalent isotropic displacement parameters (Å^2^)

**Table:**

	*x*	*y*	*z*	*U*_iso_*/*U*_eq_	Occ. (<1)
C1	0.38918 (12)	0.1439 (4)	0.6248 (5)	0.0269 (8)	
C2	0.35730 (13)	0.0484 (4)	0.5342 (5)	0.0292 (8)	
H2	0.3602	−0.0538	0.5477	0.035*	
C3	0.32268 (13)	0.1047 (4)	0.4283 (5)	0.0373 (9)	
H3	0.3014	0.0421	0.3693	0.045*	
C4	0.31924 (13)	0.2591 (4)	0.4085 (6)	0.0371 (9)	
C5	0.34829 (13)	0.3498 (4)	0.4998 (5)	0.0345 (9)	
H5	0.3453	0.4522	0.4880	0.041*	
N1	0.28526 (15)	0.3204 (5)	0.2816 (7)	0.0721 (15)	
N2	0.42542 (12)	0.0961 (4)	0.7215 (4)	0.0343 (8)	
N3	0.38170 (11)	0.2922 (3)	0.6083 (4)	0.0298 (7)	
N4	0.47684 (11)	0.3034 (3)	−0.0187 (4)	0.0267 (7)	
O1	0.2679 (4)	0.2322 (7)	0.1676 (14)	0.105 (4)	0.737 (19)
O2	0.2783 (4)	0.4550 (6)	0.280 (2)	0.078 (4)	0.737 (19)
O1'	0.2456 (5)	0.258 (2)	0.279 (4)	0.089 (7)	0.263 (19)
O2'	0.2901 (9)	0.4556 (11)	0.254 (6)	0.050 (6)	0.263 (19)
O3	0.39040 (9)	0.3227 (3)	0.0338 (4)	0.0330 (6)	
O4	0.44135 (9)	0.2156 (3)	0.2645 (3)	0.0325 (6)	
O5	0.44161 (9)	0.4796 (3)	0.2125 (3)	0.0336 (6)	
S1	0.43441 (3)	0.33299 (9)	0.13275 (11)	0.0231 (2)	
H4B	0.5038 (10)	0.282 (4)	0.038 (5)	0.040 (12)*	
H4A	0.4797 (12)	0.384 (3)	−0.087 (4)	0.040 (12)*	
H3A	0.4008 (12)	0.356 (4)	0.663 (5)	0.043 (12)*	
H2B	0.4319 (13)	0.001 (2)	0.732 (6)	0.046 (12)*	
H2A	0.4435 (13)	0.154 (3)	0.787 (5)	0.051 (14)*	

### Atomic displacement parameters (Å^2^)

**Table:**

	*U*^11^	*U*^22^	*U*^33^	*U*^12^	*U*^13^	*U*^23^
C1	0.0336 (18)	0.0247 (18)	0.0225 (17)	−0.0018 (15)	0.0077 (16)	−0.0052 (15)
C2	0.038 (2)	0.0201 (17)	0.0297 (19)	−0.0061 (15)	0.0054 (17)	−0.0005 (16)
C3	0.034 (2)	0.039 (2)	0.039 (2)	−0.0128 (17)	−0.0037 (18)	0.0011 (19)
C4	0.0303 (19)	0.039 (2)	0.042 (2)	−0.0001 (17)	0.0015 (17)	0.0127 (19)
C5	0.038 (2)	0.0248 (18)	0.040 (2)	0.0018 (16)	0.0134 (18)	0.0053 (17)
N1	0.046 (2)	0.070 (3)	0.100 (4)	−0.007 (2)	−0.023 (3)	0.039 (3)
N2	0.0415 (19)	0.0261 (17)	0.0353 (19)	−0.0004 (15)	−0.0064 (15)	−0.0048 (15)
N3	0.0382 (18)	0.0210 (15)	0.0303 (17)	−0.0046 (13)	0.0038 (14)	−0.0074 (14)
N4	0.0323 (17)	0.0268 (16)	0.0211 (15)	0.0009 (13)	−0.0010 (13)	0.0003 (13)
O1	0.097 (7)	0.089 (5)	0.129 (7)	−0.034 (4)	−0.080 (6)	0.036 (4)
O2	0.055 (6)	0.073 (5)	0.105 (8)	0.032 (3)	0.014 (5)	0.029 (4)
O1'	0.062 (10)	0.099 (11)	0.106 (14)	0.002 (9)	−0.043 (9)	0.003 (11)
O2'	0.032 (10)	0.050 (10)	0.068 (11)	0.023 (6)	0.008 (10)	0.026 (7)
O3	0.0326 (13)	0.0323 (14)	0.0340 (14)	−0.0008 (11)	−0.0074 (12)	−0.0047 (12)
O4	0.0407 (15)	0.0289 (13)	0.0277 (13)	−0.0039 (12)	0.0010 (12)	0.0047 (11)
O5	0.0415 (14)	0.0245 (13)	0.0349 (14)	0.0030 (12)	−0.0083 (12)	−0.0085 (11)
S1	0.0293 (4)	0.0191 (4)	0.0210 (4)	−0.0007 (3)	−0.0026 (4)	−0.0015 (3)

### Geometric parameters (Å, º)

**Table:**

C1—N2	1.317 (5)	N1—O2'	1.243 (8)
C1—N3	1.357 (4)	N1—O1'	1.246 (8)
C1—C2	1.411 (5)	N1—O1	1.257 (6)
C2—C3	1.348 (5)	N2—H2B	0.884 (18)
C2—H2	0.9300	N2—H2A	0.877 (18)
C3—C4	1.402 (6)	N3—H3A	0.886 (19)
C3—H3	0.9300	N4—S1	1.657 (3)
C4—C5	1.338 (6)	N4—H4B	0.889 (18)
C4—N1	1.449 (6)	N4—H4A	0.890 (18)
C5—N3	1.340 (5)	O3—S1	1.440 (3)
C5—H5	0.9300	O4—S1	1.451 (3)
N1—O2	1.228 (6)	O5—S1	1.460 (2)
			
N2—C1—N3	119.3 (3)	O1'—N1—O1	50.3 (11)
N2—C1—C2	123.4 (3)	O2—N1—C4	119.1 (8)
N3—C1—C2	117.3 (3)	O2'—N1—C4	114.0 (14)
C3—C2—C1	120.3 (3)	O1'—N1—C4	115.3 (10)
C3—C2—H2	119.8	O1—N1—C4	116.7 (5)
C1—C2—H2	119.8	C1—N2—H2B	122 (2)
C2—C3—C4	118.9 (4)	C1—N2—H2A	124 (3)
C2—C3—H3	120.5	H2B—N2—H2A	114 (3)
C4—C3—H3	120.5	C5—N3—C1	122.9 (3)
C5—C4—C3	120.7 (4)	C5—N3—H3A	116 (3)
C5—C4—N1	119.8 (4)	C1—N3—H3A	120 (3)
C3—C4—N1	119.4 (4)	S1—N4—H4B	109 (3)
C4—C5—N3	119.6 (3)	S1—N4—H4A	108 (2)
C4—C5—H5	120.2	H4B—N4—H4A	112 (3)
N3—C5—H5	120.2	O3—S1—O4	114.22 (15)
O2—N1—O2'	17.8 (19)	O3—S1—O5	112.50 (15)
O2—N1—O1'	107.6 (12)	O4—S1—O5	111.59 (15)
O2'—N1—O1'	122.5 (12)	O3—S1—N4	105.26 (16)
O2—N1—O1	123.8 (8)	O4—S1—N4	103.93 (15)
O2'—N1—O1	123.5 (19)	O5—S1—N4	108.63 (15)
			
N2—C1—C2—C3	−175.9 (4)	C5—C4—N1—O2'	8 (2)
N3—C1—C2—C3	3.6 (5)	C3—C4—N1—O2'	−169 (2)
C1—C2—C3—C4	0.6 (6)	C5—C4—N1—O1'	−141.5 (16)
C2—C3—C4—C5	−3.2 (6)	C3—C4—N1—O1'	41.3 (16)
C2—C3—C4—N1	174.0 (4)	C5—C4—N1—O1	162.1 (8)
C3—C4—C5—N3	1.4 (6)	C3—C4—N1—O1	−15.1 (9)
N1—C4—C5—N3	−175.7 (4)	C4—C5—N3—C1	3.1 (6)
C5—C4—N1—O2	−11.3 (11)	N2—C1—N3—C5	174.0 (3)
C3—C4—N1—O2	171.4 (9)	C2—C1—N3—C5	−5.5 (5)

### Hydrogen-bond geometry (Å, º)

**Table:**

*D*—H···*A*	*D*—H	H···*A*	*D*···*A*	*D*—H···*A*
N2—H2*B*···O4^i^	0.88 (2)	1.98 (2)	2.861 (4)	176 (4)
N2—H2*A*···N4^ii^	0.88 (2)	2.18 (2)	3.044 (4)	169 (4)
N3—H3*A*···O5^iii^	0.89 (2)	1.91 (2)	2.766 (4)	163 (4)
N4—H4*B*···O4^iv^	0.89 (2)	2.20 (2)	3.073 (4)	166 (3)
N4—H4*A*···O5^v^	0.89 (2)	2.20 (2)	2.960 (4)	143 (3)
C2—H2···O3^i^	0.93	2.57	3.469 (4)	163
C3—H3···O2^vi^	0.93	2.46	3.328 (13)	155
C5—H5···O3^iii^	0.93	2.41	3.187 (4)	141

Symmetry codes: (i) *x*, −*y*, *z*+1/2; (ii) *x*, *y*, *z*+1; (iii) *x*, −*y*+1, *z*+1/2; (iv) −*x*+1, *y*, −*z*+1/2; (v) *x*, −*y*+1, *z*−1/2; (vi) −*x*+1/2, *y*−1/2, *z*.
